# The relationship between the gut microbiome and resistance training: a rapid review

**DOI:** 10.1186/s13102-023-00791-4

**Published:** 2024-01-02

**Authors:** Adam Wagner, Kateřina Kapounková, Ivan Struhár

**Affiliations:** 1 Department of Sport Performance and Exercise Testing Promotion, Faculty of Sport Studies, Masaryk University, Kamenice 5, 625 00 Brno, Czech Republic; 2 Department of Physical Activities and Health Sciences, Faculty of Sport Studies, Masaryk University, Kamenice 5, 625 00 Brno, Czech Republic

**Keywords:** Gut microbiome, Resistance training, Diversity (list three to ten pertinent keywords specific to the article yet reasonably common within the subject discipline.)

## Abstract

The human gut microbiome is attracting increasing attention because of its overall effect on health. Several reviews have investigated the impact of physical activity on the gut microbiome; however, these predominantly concentrate on either endurance or a combination of physical activities. This study aims to describe the effect of resistance or strength training on the gut microbiome of a human population. This rapid review follows the guidelines of the Cochrane Rapid Reviews Guidance along with PRISMA. A review of the literature was carried out using articles indexed by PubMed, Scopus, and Web of Science published in the last 12 years. None of the seven studies included find significant change in the gut microbiome in terms of bacterial taxa composition or overall diversity, though the results show that resistance training might decrease the zonulin level and increase mucin production and thereby reduce inflammation in the gut. Interestingly, two studies point to a gut-muscle axis connection and this is discussed in our paper. However, due to the small number of existing studies and certain methodological disagreements, it was hard to find a consensus on the relationship between the gut microbiome and resistance training.

## Highlights

The links between resistance training and the gut microbiome have yet to be fully elucidated.

Resistance training may reduce inflammation through modulation of level zonulin and mucin.

The gut microbiome may influence performance in resistance training through the gut-muscle axis connection.

## Background

The gut microbiome forms a large part of the total human microbiota, which has been shown in recent years to play a crucial role in maintaining human health [1]. Its content consists of more than 2,000 bacterial strains, with 90% represented by strains of *Bacteroidetes* and *Firmicutes* [1]. The gut microbiota begins to form before birth. Several studies have confirmed the presence of specific strains of bacteria in the meconium [2]. There is a relationship between the oral microbiota of the mother and the placental microbiota [3]. for example, women with heavy periodontitis have a higher risk of adverse pregnancy outcomes, such as preterm birth and low birth weight [3].

The method of birth also affects the development and composition of the gut microbiota [2]. With a cesarean section, there is no natural delivery of the vaginal and intestinal microbiota from the mother. The gut microbiome of these newborns differs in composition; instead of the genera *Bifidobacterium* and *Lactobacillus*, there is a significant proportion of the phylum *Proteobacteria* [4]. As a result, babies born in this way take several months to acquire a normal gut microbiome. Furthermore, children born by cesarean section are much more likely to develop allergies, autoimmune diseases, asthma, and a generally poorer resistance of the human body to pathogens [5].

Several essential functions have been assigned to the gut microbiome [6]. These functions include fermentation of microbiota-accessible carbohydrates into absorbable metabolites, formation of signaling molecules, protection against pathogens, the strengthening of the intestinal barrier, and production of vitamins [7–11]. Most of these functions are closely connected to human physiology. For example, short chain fatty acids production (SCFA) is the main source of energy for intestinal epithelial cells and can make up as much as 6–10% of daily calorie intake [7]. Furthermore, it can also reduce the occurrence of fat in the blood and energy storage in the adipose tissue through activation of AMP (AMP – activated protein kinase) which is involved in the regulation of fatty acids [12]. It can also reduce inflammatory reactions in the body, exerting an anti-cancer effect and contributing to the suppression of cancer cells [13, 14].

The role played by physical activity (PA) in human health has been known for a long time. There are countless benefits of PA, for example reducing the risk of chronic diseases, helping to manage weight, improving mental health, strengthening muscle and bones, improving sleep quality, lowering blood pressure, and maintaining blood sugar level. There is a difference between the benefits of each type of physical activity. Resistance training, which is the main subject of this systematic review, has the following benefits: reversing muscle loss, recharging resting metabolism, reducing body fat, facilitating physical function, resisting type 2 diabetes, improving cardiovascular health (resting blood pressure, blood lipid profiles, vascular condition), increasing bone mineral density, enhancing mental health and reversing aging factors [15–23].

The relationship between the gut microbiota and PA was first examined in animal samples and later in humans. These studies highlighted the ability of PA to modify the composition of the gut microbiome [1, 24]. According to current sources, regular PA increases the diversity of the intestinal microbiota and the number of beneficial bacteria, particularly those that produce SCFA. PA also leads to better intestinal blood flow and improved intestinal motility, thereby accelerating digestion passage through the intestinal tract and thereby serving to prevent constipation and associated problems [25]. There have already been several systematic reviews focusing on the effect of exercise and physical activity on the gut microbiome, though none have focused exclusively on the effect of resistance training [26–31]. Among the published reviews, Ramos et al. (2022) conducted a systematic review focusing on the effects of PA on the gut microbiome of older adults, Bonomini – Gnutzmann et al. (2022) summarized the effect of intensity and duration of exercise on gut microbiota in humans, Dorelli et al. (2021) described the influence of PA on gut microbiota composition independently of diet, Cataldi et al. (2022) devoted their attention to the difference between the effect of PA on the gut microbiome in healthy and unhealthy subjects, and Zheng et al. (2022) collected knowledge about the influence of physical exercise on obesity and type 2 diabetes. There have also been other reviews that focused on the overall effect of PA and that either used only specific study designs or also included animal studies [26, 30].

The main aim of this rapid review is to summarize the results of human studies that examine the effect of resistance training on the gut microbiome of a healthy or unhealthy population to understand whether resistance training has the potential to positively modulate the gut microbiome.

## Materials and methods

### Data sources and search strategy

This rapid review followed the guidelines of the Cochrane Rapid Reviews Guidance along with the Preferred Reporting Items for Systematic Reviews and Meta-Analyses—PRISMA 2020 [32–34]. The method of rapid review was chosen because it sped up the process of the traditional systematic review guidelines. The author poses a primary research question and then transforms it to the population, intervention, comparison, outcomes, and study design system (PICOs).

A review of the literature was carried out using articles indexed by PubMed, Scopus, and Web of Science published from 1 January 2010 to 31 October 2022. The following terms were used for the search: strength training, resistance training, bodybuilding, weightlifting, weight training, strength exercise microbiota, microbiome, microflora, intestinal, gut, physical activity, fitness, sports. All descriptors were searched using Boolean operators to maximize search quality as follows: (“gut microbiome” OR “gut microbiota” OR “gut microflora” OR “gut microbes” OR “intestinal microbiome” OR “intestinal microbiota” OR “intestinal microflora” OR “intestinal microbes”) AND (“strength training” OR “resistance training” OR “strength exercise” OR “weight training” OR “weightlifting” OR “bodybuilding”). The search for PubMed was a combination of Boolean operators and the MeSH database.

### Eligibility criteria

Our inclusion criteria were: (i) studies on human subjects, (ii) English-language research articles, (iii) the studies were experimental studies, randomized controlled trials, quasi-experimental studies, including observational and cohort studies. The exclusion criteria were: (i) non-English studies, (ii) animal studies, (iii) articles with no full-text available. The selection criteria are summarized in the population, intervention, comparison, outcomes and study design system (PICOs) (Table 1).

**Table 1:** PICOs criteria for inclusion and exclusion of studies

Parameter	Inclusion Criteria	Exclusion Criteria
Population	Healthy and unhealthy population, from sedentary lifestyle to professional athletes, no age limit	Animals, Subjects who take or have taken (before intervention) antibiotics, pre/probiotics or symbiotics
Intervention	Resistance training intervention, or measurement in specific strength athletes	Any kind of endurance PA/PE, combined PA
Comparison	Different type of physical activity (e.g., endurance, combined, HIIT) or control group without intervention	Absence of any kind of comparison
Outcomes	Alpha and beta diversities Taxonomical composition Microorganism abundance	Lack of measurements which are included in inclusion criteria
Study type	Observational studies Intervention studies	Systematic reviews, Meta-analysis, case study

### Study selection and data extraction

**Fig. 1:**
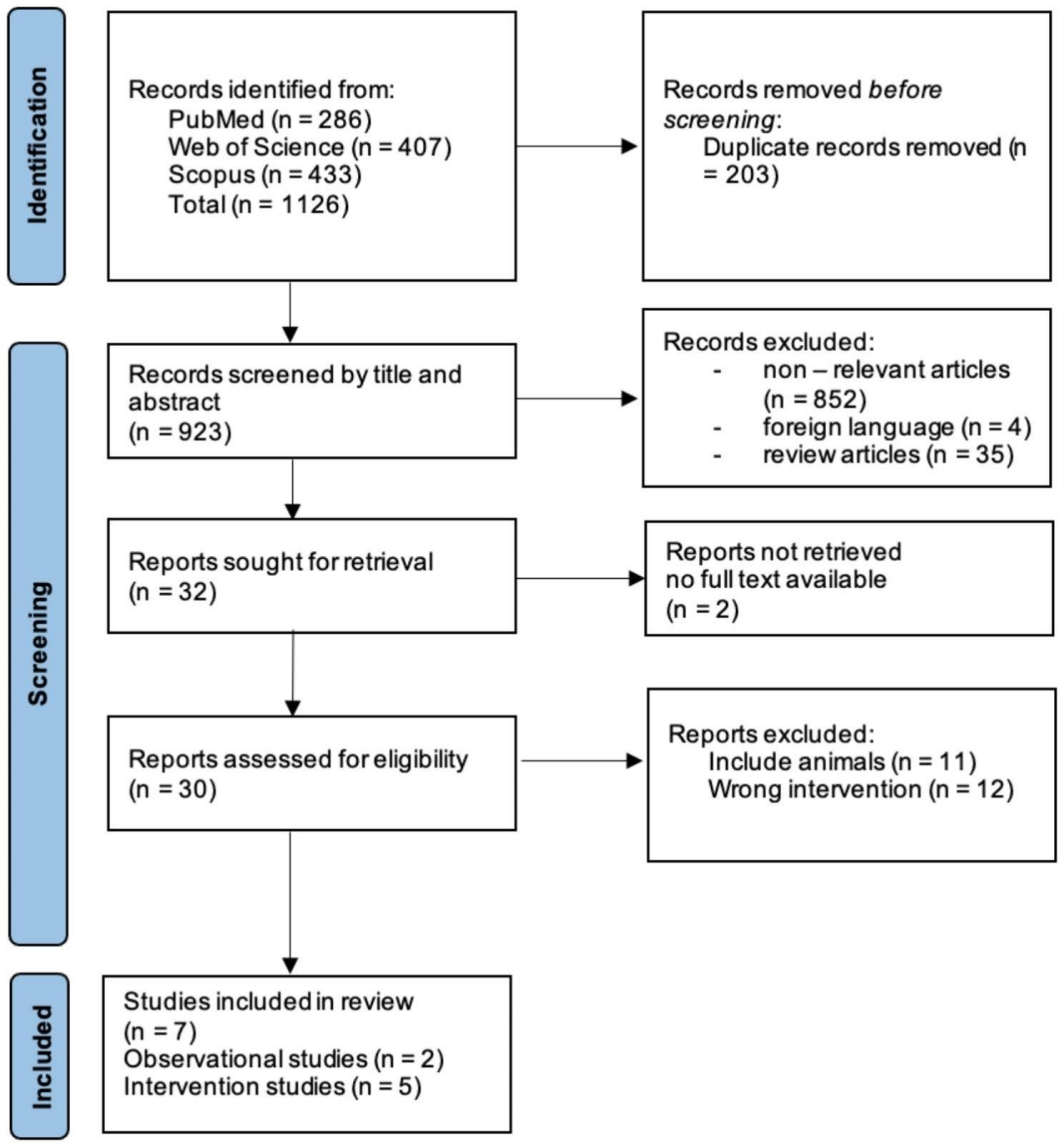
PRISMA flow diagram showing the selection process

Duplicates were first excluded after the search studies were inserted into Rayyan systematic review software [35]. The title and abstract were then scanned based on keywords and the context of the study. Thirty studies were included in the final selection as meeting the eligibility criteria. The selection process is displayed in a PRISMA flow diagram (Fig. 1).

### Risk of Bias

A risk of bias was performed to determine the study qualities included in this review. A modified version of the Jadad Scale was used to assess the quality of randomized controlled trials (Table 2). This scale contains five items and the total score for each study fell within a range from 0 to 5 [36]. The Risk of Bias in Non-Randomized Studies of Interventions (ROBINS-I) tool was used to assess quality in non-randomized studies of intervention (Table 3). This tool contains seven items and an overall score for each article resulting from detailed appraisal [37]. The risk of bias in cross-sectional articles was evaluated by the JBI Critical Appraisal Checklist for Analytical Cross-Sectional Studies (Table 4). This checklist contains eight items and an overall appraisal [38].

**Table 2:** The modified version of the Jadad Scale

Authors	Was the Treatment Randomly Allocated?	Was the Randomization Procedure Described and Was it Appropriate?	Was There a Description of Withdrawals and Dropout?	Was There a Clear Description of the Inclusion/Exclusion Criteria?	Were the Methods of Statistical Analysis Described?	Jadad Score (0–5)
Moore et al., 2022 [39]	Yes	Yes	Yes	Yes	Yes	5
Moitinho-Silva et al., 2021 [40]	Yes	Yes	No	Yes	Yes	4

**Table 3:** 1 ROBINS-I.

Study	Bias Due to Confounding	Bias in Selection of Participants in the Study	Bias in Classification / Measurement of Intervention	Bias Due to Deviations from Intended Interventions	Bias Because of Missing Data	Bias in Measurement of Outcomes	Bias in Selection of the Reported Result	Overall
Bycura et al., 2021 [41]	Low	Low	Low	Moderate	Moderate	Low	Low	Moderate
Morita et al., 2019 [42]	Low	Low	Low	Moderate	Moderate	Moderate	Low	Moderate
Aaman et al., 2020 [43]	Low	Moderate	Low	Moderate	High	Moderate	Moderate	Moderate

**Table 4:** JBI Critical Appraisal Checklist for Analytical Cross-Sectional Studies

Study	Were the Criteria for Inclusion in the Sample Clearly Defined?	Were the Study Subjects and the Setting Described in Detail?	Was the Exposure Measured in a Valid and Reliable Way?	Were Objective, Standard Criteria Used for Measurement of the Condition?	Were Confounding Factors Identified?	Were Strategies to Deal with Confounding Factors Stated?	Were the Outcomes Measured in a Valid and Reliable Way?	Was Appropriate Statistical Analysis Used?	Overall Appraisal (Included/Excluded)
Szurkowska et al., 2021 [44]	Y	Y	Y	Y	N	N	Y	Y	I
Jang et al., 2019 [45]	Y	Y	Y	Y	Y	N	Y	Y	I

## Results

The main characteristics of the included studies are shown in Table 5, and the findings of the included studies on microbial changes, diversity differences, and other intestinal changes are presented in Table 6.

### Summary of gut microbiome responses to resistance training

Six studies were looking for microbial changes in groups that were performing or related to resistance training. Moore et al. (2022) sought changes in the gut microbiota in healthy adults who performed full-body resistance training twice a week for 6 weeks. Changes in five microbial genera were evaluated: *Bacillus*, *Lactobacillus*, *Clostridium*, *Streptococcus*, *Bifidobacterium.* The results show that resistance training did not affect the microbial composition in terms of microbial taxonomical changes [39]. The same results were observed by Bycura et al. (2021) when they compared changes induced by resistance exercise (RE) and cardiorespiratory exercise (CRE). They found some significant changes in the CRE group, while they found no significant changes in taxonomical composition in the RE group. They did, however, find difference between individuals who perform a high 3RM squat and those who performed a low 3RM squat. Those with a higher 3RM squat had a significantly greater abundance of the genera *Ruminococcus*, *Lachnospiraceae*, *Turicibacter* and *Clostridium.* In contrast, those who had a lower 3RM squat had a significantly greater abundance of the genera *Siccibacter*, *Bacteroides*, *Bacteroides B*, *Alistipes*, and *Oscillibacter* [41]. Morita et al. (2019) also compared the effect of strength and endurance training intervention in healthy sedentary elderly women. A significant change was found only in the genus *Clostridium cluster IX* after 1-hour upper-body resistance training per week for 12 weeks [42]. Aaman et al. (2019) did not find any difference in microbial composition after 12 weeks resistance training in patients with cirrhosis. Interestingly, there was a difference in microbial composition in patients who did not improve their muscle strength in the phylum *Proteobacteria* (*Piscirickettsiaeae*, *Hyphomonadaceae*, *Caulobacterales*) compared to those who improved muscle strength [43]. Differences between young healthy men and bodybuilders were observed by Szurkowska et al. (2019). The overall results show that the group bodybuilders did not show any significant difference in taxonomical composition [47]. In contrast, Jang et al. (2019) found an increased abundance of *Faecalibacterium*, *Sutterella*, *Clostridium*, *Haemophilus* and *Eisenbergiella* and a decreased abundance of *Bifidobacterium* and *Parasutterella* in comparison with the control group [45].

Four studies also evaluated changes in the diversity of the gut microbiome. None of the studies found any difference regarding alpha diversity due to resistance training [39, 41, 43, 46]. The same results were also found for beta diversity [39, 41, 46].

Some studies also looked for other changes related to the gut [39, 44]. Moore et al. (2022) considered changes in SCFA production, mucin biosynthesis, mucin degradation, LPS, and zonulin levels. They reported significantly higher mucin biosynthesis and a decrease in zonulin level. Szurkowska et al. (2019) observed higher fecal pH in bodybuilders.

### Impact of training status on human microbiota and health

In light of the studies reviewed, the gut microbiota of trained individuals appears to exhibit a distinct composition and functional capacity compared to untrained subjects. For instance, Moore et al. (2022) and Bycura et al. (2021) noted no significant changes in microbial taxa due to resistance training in healthy adults and students, respectively, although differences were noted based on the individuals’ squat strength, suggesting a possible link between muscle strength and gut microbiota composition [39, 41]. These findings suggest that resistance training may exert subtle yet potentially meaningful influences on gut health, particularly concerning mucin biosynthesis and zonulin levels, markers for gut barrier integrity and permeability [39]. Conversely, initiating resistance training in untrained individuals may lead to early shifts in gut microbiota composition, as observed in studies involving sedentary or unhealthy populations. Aamann et al. (2020) reported no change in the overall microbial composition after resistance training in patients with cirrhosis, yet those who did not improve muscle strength exhibited a different microbial profile, underscoring the potential influence of physical strength gains on gut microbiota [43]. A comparative analysis between trained and untrained individuals reveals that while the direction of microbial shifts appears positive in both groups, the extent and nature of these shifts vary with the individuals’ baseline fitness levels and training status. This is further exemplified by Jang et al. (2019), who observed an increased abundance of certain bacterial taxa such as *Faecalibacterium* in bodybuilders, potentially linked to dietary protein intake and its effect on gut pH [45].

**Table 5:** Characteristics of the included studies

Authors	Sample	Exercise protocol	Duration	GM Analysis System
Moore et al., 2022 [39]	subjects 51–78 years (n = 14)	full-body resistance training 2xwk (5 exercises, 3 sets and 10–12 repetitions, RPE 7–9)	6 weeks	
Moitinho-Silva et al., 2021 [40]	healthy physically inactive subjects 21–41 years (n = 36), aerobic exercise (AE) (n = 12), strength exercise (SE) (n = 13) with control (n = 11), elite athletes for comparison (n = 13)	AE group: 30 min 3xwk running SE group: 30 min 3xwk whole-body hypertrophy strength training	6 weeks	16 S rRna GA V1/V2 region
Bycura et al., 2021 [41]	healthy students 18–33 years (n = 56), cardiorespiratory exercise (CRE) (n = 28), resistance exercise (RE) (n = 28)	CRE group: 1 h, 3xwk (2-day group cycling, 1-day rotating CRE activity) 60–90% HR_max_ RE group: 1 h, 3xwk full/lower/upper-body at 70–85% 1RM	8 weeks	16 S rRNA GA V4 region
Morita et al., 2019 [42]	healthy sedentary elderly women 66–75 years (n = 32), resistance exercise (RE) (n = 14), aerobic exercise (AE) (n = 18)	RE group: 1 h weekly upper-body resistance training AE group: 1 h daily brisk walking > 3 METs	12 weeks	16 S rRNA GA
Aaman et al., 2020 [43]	patients with cirrhosis (n = 34)	progressive resistance training 3xwk full-body	12 weeks	16 S rRNA GA
Szurkowska et al., 2021 [44]	young healthy men aged 22–28 years (n = 26), amateur bodybuilders (n = 11) and controls of a similar age (n = 15)	Observational study	/	PCR
Jang et al., 2019 [45]	bodybuilders (n = 15), endurance athletes (n = 15), sedentary individuals (n = 15)	Observational study	/	16 S rRna GA V3/V4 region

**Table 6:** Changes in the gut microbiome

Reference	Results				
	**Microbial changes**	**Diversity**		**Other gut-related changes**	
Moore et al., 2022 [39]	Genera: *Bacillus* ↔ *Lactobacillus* ↔ *Clostridium* ↔ *Streptococcus* ↔ *Bifidobacterium* ↔		Alpha diversity ↔ Beta diversity ↔		SCFA production ↔ Mucin biosynthesis ↑ Mucin degradation ↔ LPS ↔ Zonulin ↓
Moitinho-Silva et al., 2021 [40]	-		Alpha diversity ↔ Beta diversity ↔		-
Bycura et al., 2021 [41]	↔ in RE group Only individuals who experience high 3RM squats change had ↑ genera *Ruminococcus*, *Lachnospiraceae*, *Turicibacter* and *Clostridium* The individuals who experience low 3RM squats change had ↑ *Siccibacter*, *Bacteroides*, *Bacteroides B*, *Alistipes*, *Oscillibacter*		Alpha diversity ↔ Beta diversity ↔		-
Morita et al., 2019 [42]	Genera *Bacteroides* ↔ *Prevotella* ↔ *Bifidobacterium* ↔ *Lactobacillales* ↔ *Clostridium cluster XVIII* ↔ *Clostridium cluster XI* ↔ *Clostridium cluster IX* ↑ *Clostridium cluster IV* ↔ *Clostridium subcluster XIVa* ↔		-		-
Aaman et al., 2020 [43]	Patients who did not improve their muscle strength had ↑ *Proteobacteria* (*Piscirickettsiaeae*, *Hyphomonadaceae*, *Caulobacterales*)		Alpha diversity ↔		-
Szurkowska et al., 2021 [44]	*Bifidobacterium spp.* ↔ *Bacteroides spp.* ↔ *Akkermansia muciniphila* ↔ *Faecalibacterium prasnitzii* ↔		-		Fecal pH ↑ in bodybuilders
Jang et al., 2019 [45]	Bodybuilders: *Faecalibacterium* ↑ *Sutterella* ↑ *Clostridium* ↑ *Haemophilus* ↑ *Eisenbergiella* ↑ *Bifidobacterium* ↓ *Parasutterella* ↓		-		-

## Discussion

To our knowledge, this is the first systematic review to examine how resistance training affects the human gut microbiota. According to the studies analyzed, resistance training did not affect the gut microbiome in terms of microbial composition or general diversity. Only Morita (2019) found a significant increase in *Clostridia IX*, which was flagged as irrelevant due to the low overall representation of this cluster. Some interesting results with a connection between resistance training and gut microbiome or gut health were, however, observed in other studies which are discussed below.

### Resistance training and its impact on gut barrier function

In addition to changes in gut microbiome composition, some studies have also examined gut-related changes due to resistance training [39, 44]. One of these changes was a decrease in the level of zonulin [39]. This protein is related to the formation of tight junctions between intestinal epithelial cells. Based on current knowledge, a higher level of zonulin is an indicator of ‘leaky gut’ syndrome [47, 48]. A decrease in the zonulin level was also observed after six weeks of combined endurance and resistance training in type 2 diabetes patients [49]. A significant increase in mucin biosynthesis was observed in the same study. Mucin is a protein that plays a critical role in human mucosal immunity [50]. Gut microbes directly affect goblet cell function through the local release of bioactive factors generated by activated epithelium cells or underlying lamina propria cells. Gut microbes can also regulate mucin production by activating different signaling paths and secretory elements [51]. For this reason, these results provide the information that resistance training can affect intestinal barrier integrity through moderate, though apparently meaningful, changes in the composition of gut microbes responsible for mucin biosynthesis. Although this is an interesting theory, it needs to be evaluated by future studies.

### Potential gut-muscle axis modulation through strength training

**Fig. 2:**
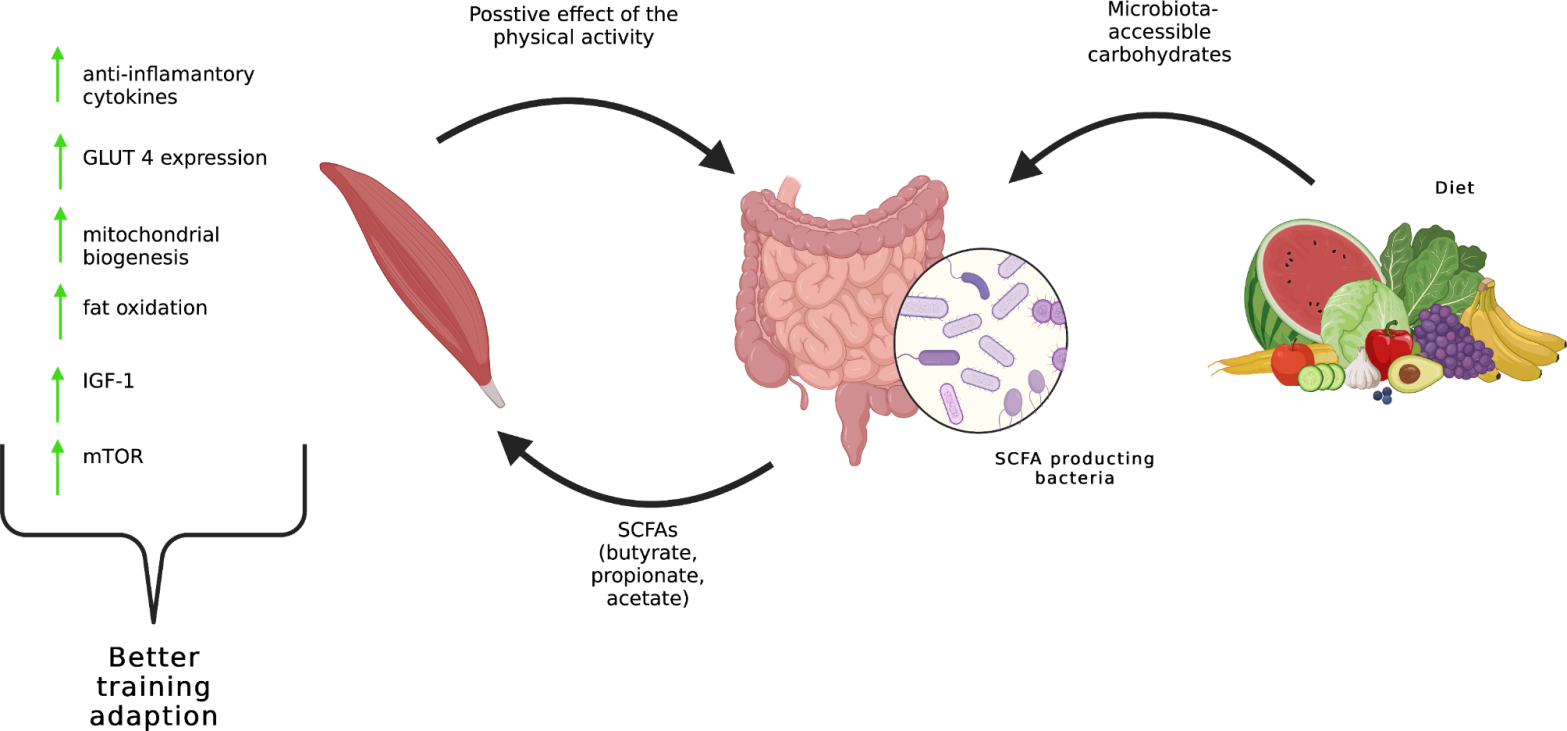
The relationship between diet and gut-muscle axis

A somewhat surprising relationship was the difference in gut microbiome composition in those who improved their 3RM squat after 8 weeks of resistance training [41]. Subjects who experienced a higher 3RM squat had a significantly larger amount of the genera *Ruminococcus*, *Lachnospiraceae*, *Turicibacter* and *Clostridium.* Those with lower 3 RM squat had a significantly greater abundance of the genera *Siccibacter*, *Bacteroides*, *Bacteroides B*, *Alistipes* and *Oscillibacter.* This difference may be related to the gut-muscle axis, which was discussed in a number of the research papers [52, 53]. This axis is the connection between gut health and function and muscle health and function. It seems that the gut microbiome may influence muscle protein synthesis, mitochondrial biogenesis / function, and glycogen storage [52, 53]. Muscle protein synthesis and training adaptation seem limited under chronic inflammation, which is directly affected by the gut microbiome [54]. Many biologically active metabolites produced by intestinal bacteria can increase/decrease inflammation [55–57]. The most well-known are SCFA (butyrate, acetate, and propionate). Butyrate may play a key role in the management of cell growth and differentiation and also has anti-inflammatory effects, such as promoting antimicrobial peptide secretion, suppressing lymphocyte or granulocyte activity, enhancing intestinal barrier integrity, or decreasing pro-inflammatory cytokine production [55, 58]. *Ruminococcus*, *Lachnospiraceae* and *Clostridium* are the main butyrate producers, for which reason subjects with higher 3RM squat and greater abundance of these types of microbes may have lower body inflammation and better training adaptation [55]. SCFAs, mainly acetate, propionate, and butyrate, interact with key signaling pathways in muscle cells. For example, butyrate has been shown to activate the mTOR signaling pathway, enhancing protein synthesis in intestinal epithelial cells [59]. Furthermore, SCFAs can influence the production of IGF-1, an anabolic hormone that promotes muscle growth, with germ-free animals often exhibiting reduced serum levels of IGF-1, suggesting a link between gut microbiota and IGF-1 production [60]. In terms of inflammation, SCFAs, and particularly butyrate, possess potent anti-inflammatory properties. They can regulate immune responses by inhibiting the production of pro-inflammatory cytokines and promoting the production of anti-inflammatory cytokines through various mechanisms, including the activation of G-protein-coupled receptors and inhibition of histone deacetylase [60]. This is significant for muscle health as chronic inflammation can lead to muscle wasting. SCFAs also play a role in glucose metabolism and insulin sensitivity, which are critical for muscle function. They can enhance GLUT-4 expression and mitochondrial biogenesis, essential for energy metabolism in muscle cells, through the activation of AMP and peroxisome proliferator-activated receptor gamma coactivator-1α (PGC-1α) [61]. The overall impact of SCFAs on muscle health is beneficial, but it may vary based on their concentrations, ratios, and the individual’s overall health and dietary habits. The gut-muscle axis represents a complex system with many factors at play [62]. Figure 2 shows a correlation between the diet and the gut-muscle axis. Aaman (2019) also observed a different composition in gut microbiome in patients who did not improve their strength, though the role of these orders in human health is still unknown.

### The interplay between bodybuilding diet, resistance training, and gut environment

Szurkowska (2021) observed a significantly higher fecal pH in bodybuilders as compared to the control group. An integral part of bodybuilding is resistance training, so the difference in fecal pH might be related to the effect of training. In this case, however, the differences are related to the high-protein animal diet making up part of the bodybuilding diet. The higher fecal pH could be the consequence of proteolytic putrefactive bacteria and their ability to produce alkaline metabolites [63]. An altered gut pH may change the composition of the gut microbiome and metabolite production. A number of articles discussed the influence of aerobic physical activity on gut pH and, thereby, an altered gut microbiome population [24, 25, 29, 30]. Changes in the passage time of colonic stools result in changes in the pH within the colon which may be key to affecting the composition of the intestinal microbiota [42, 61, 64, 65]. A prolonged transit time of the colonic stool is known to limit the diversity of the intestinal microbiota, which is associated with a greater increase in pH during passage from the proximal to the distal part of the colon [66]. Aerobic exercise, such as jogging and moderate-intensity cycling, shortens bowel movements in healthy people and in middle-aged patients with chronic constipation [67, 68]. This is probably due to an increase in visceral blood levels, increased hormone release in the gastrointestinal tract, and mechanical stimulation of the abdominal muscles. Aerobic exercise further increases the fecal concentration of SCFA, which slightly lowers the pH of the large intestine [61, 65]. It will be interesting in future studies to evaluate the fecal pH in studies with resistance training if there is the potential to alter fecal pH.

### Evaluating study designs and combined training effects on gut microbiota

The next element to discuss is the design of the included studies. There are only five studies with resistance training intervention programs. The frequency of training units was from one to three per week. This information raises the question as to whether this dose of resistance training was sufficient to cause changes in the gut microbiome. The general recommendation from the World Health Organization (WHO) is 2 per week to provide health benefits. Morita (2019), in their intervention, performed resistance training only once a week and this was focused only on the trunk muscles. This design of intervention neglects the minimum dose of resistance training to promote general health benefits, so it is expected that this amount will not affect the gut microbiome [42]. It may also be a question of the “right” ratio volume and intensity of the strength training.

As we mentioned earlier, resistance training alone did not change the composition of the gut microbiome, though it may modify the composition of the intestinal microbiome in combination with high-intensity work or endurance training [49, 69, 70]. Pasini (2018) observed changes in the intestinal microbiota in individuals with type 2 diabetes after a combination of aerobic and resistance training. He focused mainly on yeast. He recorded a reduction in *Candida albicans* yeast and some other fungal species after a six-month intervention program. He also noticed a reduction in zonulin toxin which disrupts the intestinal barrier [49]. A decrease in zonulin level correlates with the findings of Moore (2022). It is possible that he may have seen a significant difference if he had also looked for changes in Candida albicans yeast [39].

### Limitations and future recommendations

It is extremely difficult to isolate the direct effect of resistance training due to the other factors that can interact with and modify the human gut microbiome (e.g., nutrition, age, type of birth, antibiotics, stress level, genetics). Not all the included studies controlled for all these factors, especially diet which is a major confounding factor. There was also a highly distinct population in the included studies, and in view of the novelty of these topics there is good reason for future studies to focus on a specific population group. Furthermore, there were highly different interventions in terms of volume, intensity, and frequency.

Future studies should aim at more randomized controlled trials and should consider the potential effects of different ratio frequency, volume and intensity in resistance training. Closer inspection is needed to consider other elements that are often overlooked – measuring the metabolomic activity of the gut and the presence of viruses, fungi and bacteriophages in the gut. In short, controlling for as many factors as possible that can affect overall results.

### Conclusions

This paper has described an initial insight into the relationship between the gut microbiome and resistance training. The results of this study underline the fact that resistance training could have some potential to modify the gut microbiome in terms of metabolomics. Specifically speaking, the decrease in the zonulin level and increase in mucin production, thereby reducing inflammation in the gut. The composition of the intestinal microbiome was not affected, though more studies are strongly recommended as the current articles may feature an incorrect ratio of volume, intensity and frequency of resistance training. Two of the included studies show a connection between muscles and the gut through the gut muscle axis and discuss the protentional benefit of butyrate production bacteria to improve training adaptation. Despite the review limitation and the small number of existing studies on this topic, these results provide a significant first step towards understanding the next protentional benefit of resistance training in the gut microbiome.

## Data Availability

The datasets used and/or analyzed during the current study are available from the corresponding author on reasonable request.
